# Improvements to mechanical response tissue analysis

**DOI:** 10.1016/j.mex.2019.10.004

**Published:** 2019-10-14

**Authors:** Lyn Bowman, Anne B. Loucks

**Affiliations:** a Department of Biological Sciences, Ohio University, Athens, OH 45701, United States; b Ohio Musculoskeletal and Neurological Institute, Ohio University, Athens, OH 45701, United States

**Keywords:** Cortical Bone Mechanics Technology, Cortical bone, Bending stiffness, Bending strength, Validation, Noninvasive

## Abstract

Cortical Bone Mechanics Technology™ (CBMT) comprises certain improvements over a previous method known as Mechanical Response Tissue Analysis (MRTA). Both methods are dynamic 3-point bending tests intended for measuring the mechanical properties of cortical bone in living people. MRTA presented a theoretical potential for direct measurement of skeletal fragility, but it had acquired a reputation for error and fallen into disuse. We found sources of error in both MRTA data collection and data analysis. We describe here the fundamentals of MRTA, the major sources of error we found in MRTA, and our innovations for avoiding them.

• Data collection at many sites across the mid-shaft of the ulna bone in the forearm.
• Parameter estimation by fitting analytical complex compliance and stiffness transfer functions to empirical complex compliance and stiffness frequency response functions.
• Optimization by selecting results from frequency response functions with the smallest deviations between fits to compliance and stiffness frequency response functions.

## Graphical abstract

**Figure:**
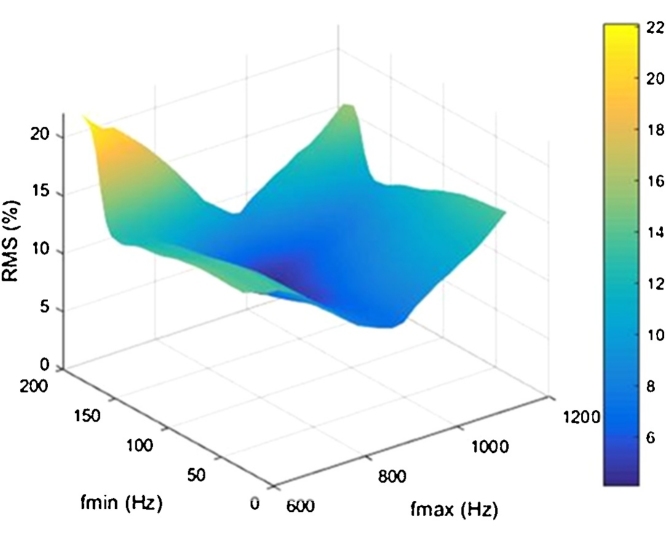

**Specification Table**

**Table:** 

Subject Area:	Engineering
More specific subject area:	Noninvasive dynamic mechanical testing of cortical bone
Method name:	Cortical Bone Mechanics Technology
Name and reference of original method:	Mechanical Response Tissue Analysis: Steele CR, Zhou LJ, Guido D, Marcus R, Heinrichs WL, Cheema C. Noninvasive determination of ulnar stiffness from mechanical response--in vivo comparison of stiffness and bone mineral content in humans. Journal of Biomechanical Engineering. 1988;110(2):87-96. doi:10.1115/1.3108423
Resource availability:	AEIOU Scientific, LLC. aeiouscientific.com

## Method details

Recently, we reported the excellent accuracy of a novel non-invasive, radiation-free method for measuring the bending stiffness and estimating the bending strength of cortical bone in ulnas of living people [1]. This method embodies certain improvements over a method known as Mechanical Response Tissue Analysis (MRTA) that had fallen into disuse [2]. MRTA was a dynamic 3-point bending test for measuring the mechanical properties of long bones in living people. It had been extensively utilized [3], but had acquired a reputation for inaccuracy [4,5] and irreproducibility [6]. Because current clinical methods do not predict fractures well [[7], [8], [9], [10]], and because MRTA presented a theoretical potential for direct measurement of skeletal fragility, we undertook to discover the major sources of error in MRTA and, if possible, to correct them. We describe here the major sources of error we found in MRTA and our innovations for avoiding them. We distinguish these innovations from MRTA by the new name Cortical Bone Mechanics Technology™ (CBMT). For those in the field of bone mechanics who are unfamiliar with frequency response analysis, we begin by describing the fundamentals of MRTA.

## Fundamentals of MRTA

### MRTA solutions for limitations of quasistatic mechanical testing

Our interest in MRTA derived from the long-known observation that quasistatic measurements of bone bending stiffness accurately predict quasistatic measurements of bone bending strength [4,[11], [12], [13]]. In QMT measurements of bone bending stiffness, a monotonically increasing bending displacement x is imposed very slowly at the middle of a long bone or bone specimen of length L while the force F required to impose the displacement is measured. Commonly, bending stiffness K_B_ = F/x is defined as the slope in the linear (i.e., elastic) range of the graphical relationship between force and displacement. To measure bending strength, the displacement is increased until the beam finally breaks. The peak moment M_peak_ = F_peak_L/4 prior to fracture is then defined as bending strength. QMT cannot be used clinically because the first step in QMT measurements of bone stiffness and strength is to remove the bone from the body.

QMT is quasistatic, because bone is a visco-elastic material. That is, the relationship between force and motion in bone depends strongly on the speed as well as the amount of displacement. Due to inertial effects, the relationship also depends strongly on the acceleration of displacement. By proceeding very slowly, QMT reduces the influences of these effects to negligible levels so that the bone’s elastic behavior (i.e., its stiffness) can be measured in isolation.

MRTA was expected to overcome the clinical limitations of QMT by applying small, rapidly oscillating forces spanning a range of audio frequencies to the skin overlying a long bone. Such dynamic mechanical testing induces viscous and inertial as well as elastic effects in both the skin and bone, and enables the mechanical properties causing those effects (i.e., damping, mass and stiffness, respectively) to be quantified in the resulting frequency-dependent vibration response. Then, the reasoning went, the bone’s strength could be accurately predicted from its stiffness.

**Fig. 1:**
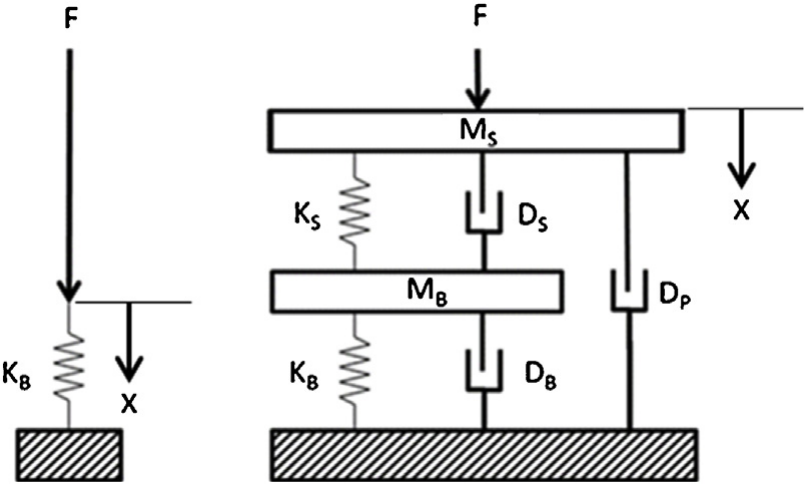
Mechanical models for QMT (left) and MRTA (right) tests. (F = force; x = displacement; M = mass, D = damping, K = stiffness; S = skin, B = bone, P = peripheral soft tissue. In QMT, skin and peripheral soft tissue are absent, and F changes so slowly that effects of M_B_ and D_B_ are negligible.

Fig. 1 contrasts the mechanical models of QMT and MRTA tests. Those familiar with mechanical dynamics will notice the presence in the MRTA model of two clusters of model parameters. One cluster is comprised of the mass, stiffness and damping of the skin (M_S_, K_S_ and D_S_) while the other is comprised of the mass stiffness and damping of the underlying bone (M_B_, K_B_ and D_B_).

The amount of force required to bend an ulna bone into the linear portion of its range of motion where K_B_ is calculated by QMT is typically more than 100 N. In MRTA, the applied static force is typically less than 20 N, and the applied oscillatory force is typically 1 N. This static force is similar to the force on one’s fingertip when pressing an elevator button, and the oscillating force is similar to the force one feels holding an electric toothbrush or electric razor.

**Fig. 2:**
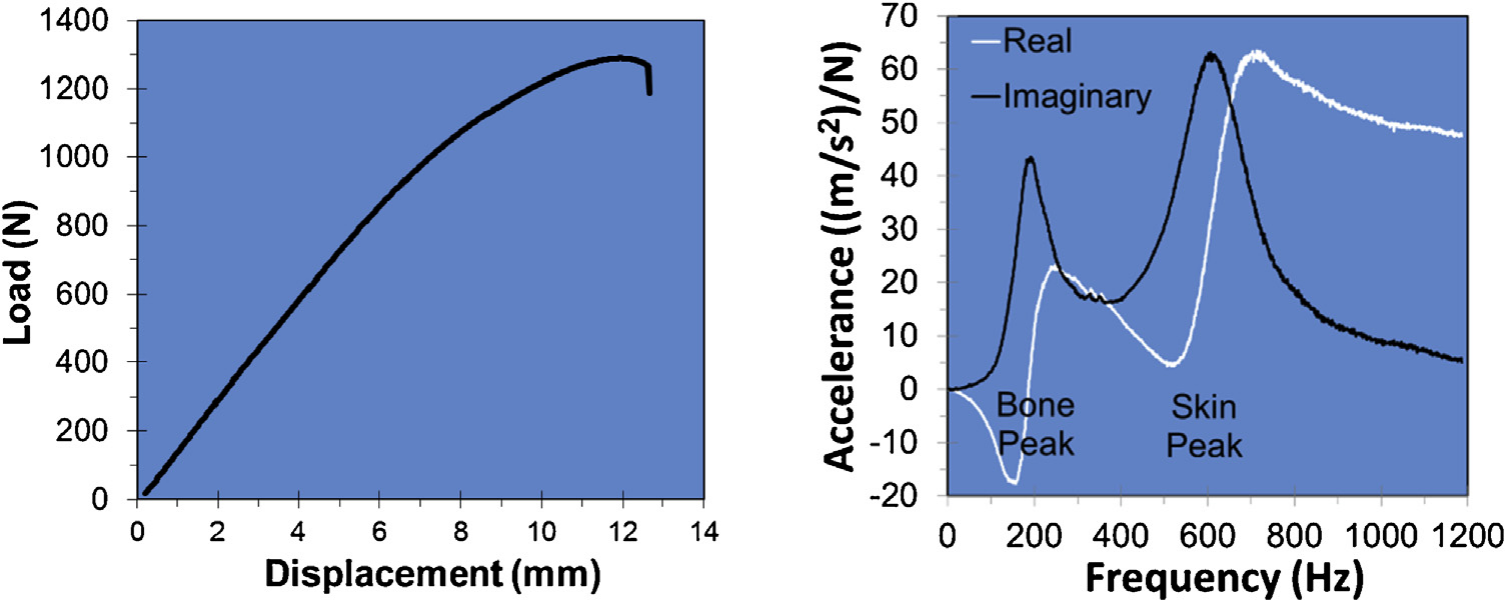
Typical QMT data (left) and MRTA data (right). Note the presence of two resonances in the MRTA data indicated by two peaks in the imaginary part and two sigmoidal curves in the real part of accelerance.

Fig. 2 shows typical QMT and MRTA data. As described above, the force and displacement data measured in QMT appear on the Y and X axes, respectively. In MRTA, the oscillatory force superimposed on the static force and the resulting acceleration of the force probe are measured, and their ratio accelerance (i.e., oscillatory acceleration divided by oscillatory force) is plotted on the Y-axis. Note that, as will be described below, this frequency-dependent response (i.e., frequency response function, FRF) has two values at every frequency: the so-called “real” and “imaginary” parts of the response.

Note, too, that the accelerance FRF in Fig. 2 varies strongly with the frequency of oscillation, which appears on the X-axis. The imaginary part of accelerance displays two peaks while the real part displays two sigmoidal curves centered near 200 and 600 Hz. The mechanical behavior at near 600 Hz is determined primarily by the mass, stiffness and damping of the skin, while the behavior at near 200 Hz is determined primarily by the mass, stiffness and damping of the underlying bone. Hence, the peaks in the imaginary part are referred to as the “skin peak” and “bone peak”, respectively. Moreover, the frequency of the skin peak increases with the static force applied to the forearm. Thus, one purpose of the static force is to separate the skin and bone peaks from one another.

### Demystification of “real” and “imaginary” parts of a frequency response function

The off-putting terms “real” and “imaginary” are nothing more an artifact of the history of mathematics when authorities in mathematics, including Rene Descartes, could not imagine how the square root of minus 1 ($\sqrt{-1}$) could possibly be useful for anything real [14]. So, such numbers were dismissed as “imaginary”. With the passage of time, $\sqrt{-1}$ was found to have many useful applications, one of which is in MRTA, but by then the derogatory name had stuck. In practice, the “real” and “imaginary” parts of a complex FRF are simply a convenient notation for keeping track of two different things.

**Fig. 3:**
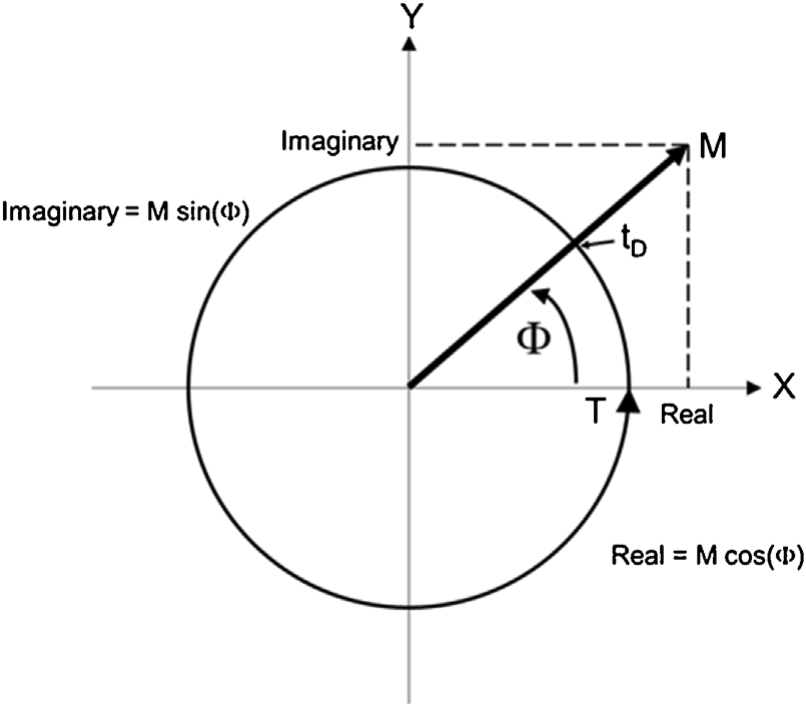
The real and imaginary parts of an oscillatory response at a single frequency f = 1/T.

To explain what these two things are, imagine I were to punch you in the nose. I would immediately be interested in two things: how *big* your response was going to be, and *when* it was going to happen. In effect, MRTA punches a person’s limb in the “nose” over and over and over again with every oscillation of force, and the limb responds with [1] some magnitude of oscillatory motion [2] some time later. In MRTA, a complex FRF contains information about the magnitudes and delays of oscillatory motions in response to oscillatory forces at different frequencies. The simple relationships between the magnitudes and times of responses, and the “real” and “imaginary” parts of those responses are illustrated in Fig. 3.

In Fig. 3, the length of time (T) for one complete cycle of the oscillatory response at one particular frequency f is defined as one *period* of the oscillation and illustrated by a circle of circumference T = 1/f. The *magnitude* M of the response is indicated by the length of an arrow pointing in a particular direction. That direction is determined by the time delay t_D_ between the peak of oscillatory force and the peak of oscillatory motion. The fraction of period T between the peak of the applied oscillatory force and the peak of the resulting oscillatory motion is defined as the *phase* Φ = t_D_/T of the response. Thus, the pair of numbers M and Φ quantify the response *at this frequency f*.

Now notice that projecting the arrow in Fig. 3 onto the X (“real”) and Y (“imaginary”) axes yields a second pair of numbers (Real = M cos (Φ) and Imaginary = M sin(Φ)) that also quantify the response. As it happens, some mathematical operations (i.e., multiplication and division) are easier to perform with the magnitude and phase expression of a complex response, while other operations (i.e., addition and subtraction) are easier to perform with the real and imaginary expression. Therefore, to facilitate calculations, the two expressions are converted back and forth for convenience.

So, what does this have to do with $\sqrt{-1}$? Because it is annoying to keep writing “real” and “imaginary” over and over again, the “real” and “imaginary” parts a and b are written as a + b $\sqrt{-1}$. And, because it is annoying to keep writing $\sqrt{-1}$ over and over again, the letter “i” or “j” is commonly used as an abbreviation for $\sqrt{-1}$. Then the “real” and “imaginary” parts of a response can be written more compactly as a + ib or as a + jb. The MRTA accelerance FRF shown in Fig. 2 is simply a compact illustration of a and b at all frequencies of oscillation at once.

### MRTA data analysis

MRTA data such as those shown in Fig. 2 are analyzed by a procedure called parameter estimation. The procedure begins by writing and solving the differential equations of motion for the mechanical model of the skin-bone system shown in Fig. 1. The solution is conveniently derived as the complex stiffness transfer function (TF) H(s) = F(s)/x(s) of the skin-bone system in the form of a 4^th^ order rational polynomial in which M_S_ is the mass of the skin [2]:

(1) $$\frac{\text{F}\left(s\right)}{x\left(s\right)}=M_{S}\frac{s^{4}+A_{3}s^{3}+A_{2}s^{2}+A_{1}s+A_{0}}{s^{2}+C_{1}s+C_{0}}$$

Substituting the complex frequency s = jω (where ω = 2πf and j = $\sqrt{-1}$) into Eq. (1) yields the real and imaginary parts of the complex stiffness TF: F/x = Real{F/x} + j Imag{F/x}, where (2)

(2a) $$\text{Real}\left\{\frac{\text{F}\left(\omega \right)}{x\left(\omega \right)}\right\}=M_{s}\frac{\left[\left(C_{0}-\omega^{2}\right)\left(\omega^{4}-A_{2}\omega^{2}+A_{0}\right)-C_{1}\omega \left(A_{3}\omega^{3}-A_{1}\omega \right)\right]}{{\left({C_{0}-\omega }^{2}\right)}^{2}+{\left(C_{1}\omega \right)}^{2}}$$

(2b) $$\text{Imag}\left\{\frac{\text{F}\left(\omega \right)}{x\left(\omega \right)}\right\}=M_{s}\frac{\left[C_{1}\omega \left(\omega^{4}-A_{2}\omega^{2}+A_{0}\right)+\left(C_{0}-\omega^{2}\right)\left(A_{3}\omega^{3}-A_{1}\omega \right)\right]}{{\left({C_{0}-\omega }^{2}\right)}^{2}+{\left(C_{1}\omega \right)}^{2}}$$

and the six coefficients in Eqs. (1) and (2) are algebraic functions of the seven parameters of the mechanical model of the skin-bone system shown in Fig. 1: (2)

(3a–d) $$A_{0}=\frac{K_{S}K_{B}}{M_{S}M_{B}}\;{\;A}_{1}=\frac{K_{B}\left(D_{S}+D_{P}\right)+K_{S}\left(D_{B}+D_{P}\right)}{M_{S}M_{B}}\;\;{\;A}_{2}=\frac{K_{S}{+K}_{B}}{M_{B}}+\frac{K_{S}}{M_{S}}+\frac{D_{S}\left(D_{B}+D_{P}\right)+D_{B}D_{P}}{M_{S}M_{B}}\;{\;A}_{3}=\frac{D_{S}{+D}_{B}}{M_{B}}+\frac{D_{S}+D_{P}}{M_{S}}$$

(3e,f) $$C_{0}=\frac{K_{S}{+K}_{B}}{M_{B}}\;C_{1}=\frac{D_{S}{+D}_{B}}{M_{B}}$$

These relationships enable the values of the parameters of the mechanical model in Fig. 1 to be obtained from the complex accelerance FRF in Fig. 2 in four steps:

(1) The complex accelerance FRF (a/F) is integrated twice to obtain the complex compliance FRF (x/F);
(2) The complex compliance FRF is inverted to obtain the complex stiffness FRF (F/x);
(3) the polynomical coefficients are obtained by fitting Eq. (1) to the complex stiffness FRF; and
(4) the parameter values are then obtained by solving the simultaneous equations in Eq. (3).

**Fig. 4:**
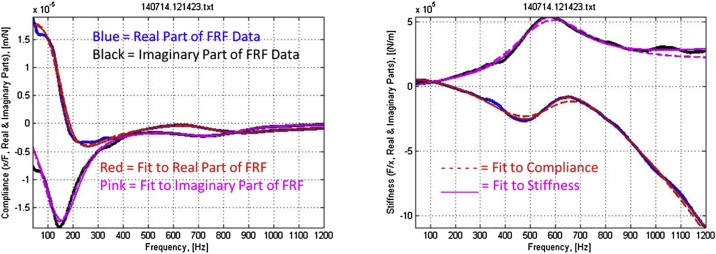
Fits of complex compliance (left) and stiffness (right) transfer functions to typical complex compliance and stiffness frequency response functions measured in MRTA tests of a forearm. Note: the X-axis begins at 40 Hz.

Alternatively, the parameter values can be obtained in three steps by skipping Step 2 and fitting the inverse of Eq. (1) to the complex compliance FRF. Fig. 4 shows fits of complex compliance and stiffness transfer functions to typical MRTA complex compliance and stiffness FRFs collected from a forearm.

## Major sources of error in MRTA

### Sources of error in MRTA data collection

**Fig. 5:**
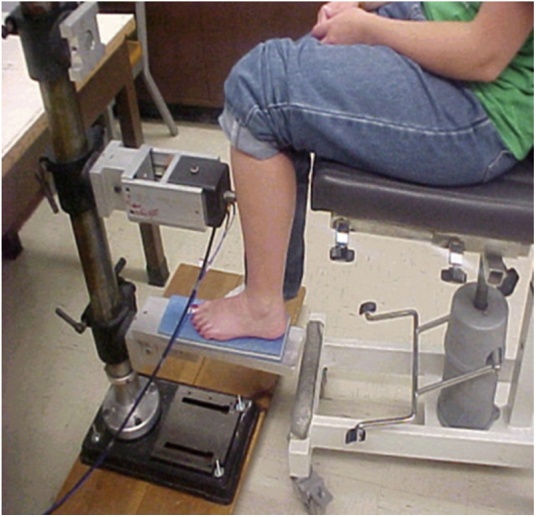
MRTA data collection from a tibia of a living human subject [15].

Fig. 5 illustrates three sources of error in MRTA data collection. First, the test subject is controlling the static force applied to the leg as she presses her leg against the force probe. Second, by the placement of her leg the subject also controls the location in the transverse direction at which the probe applies the static and oscillatory forces to the leg. As will be shown below, the resulting FRF is acutely sensitive to the placement of the probe, *and it is impossible for either the subject or the operator to know where to put it.* Third, the bone being tested in Fig. 5 is the tibia. Bending tests are highly sensitive to end conditions, and the tibia is not well supported by a mechanical ground at either end.

**Fig. 6:**
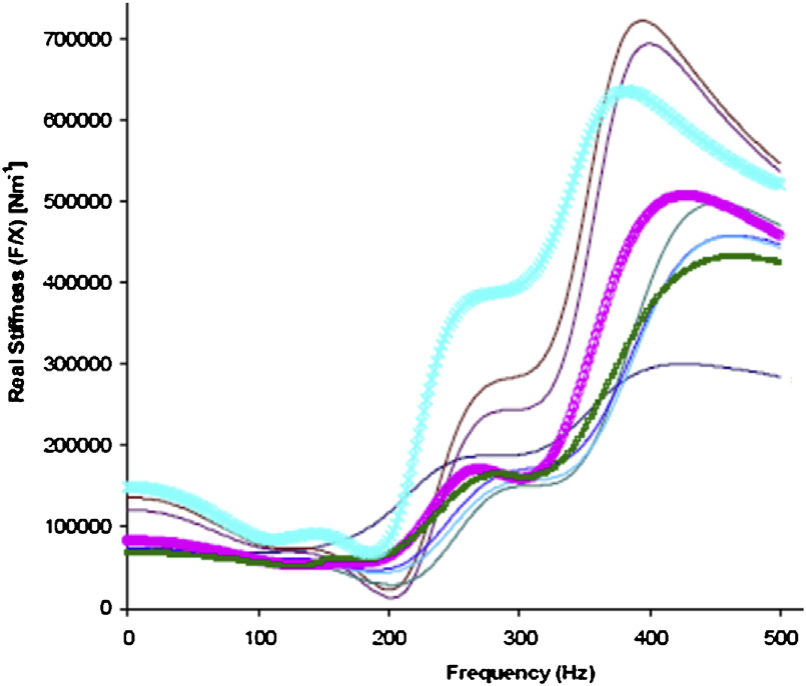
Real parts of tibia stiffness FRFs collected in the manner illustrated in Fig. 5 [6].

The property of a long bone that accurately predicts it’s bending strength is flexural rigidity (EI = K_B_ L^3^/48, where E = the elastic modulus of bone material, I = the cross-sectional moment of inertia of the bone’s geometry at the mid-span, and L = length of the bone). Fig. 6 shows the real parts of complex stiffness FRFs collected in the manner illustrated in Fig. 5 [6]. The nine repeated measurements were collected within a few minutes from a single leg. The resulting estimates of tibia EI calculated from tibial length and MRTA measurements of tibia K_B_ ranged from 143 to 653 Nm^2^ with a mean of 320 Nm^2^ and a standard deviation of 199 Nm^2^ for a coefficient of variation CV of 62%.

**Fig. 7:**
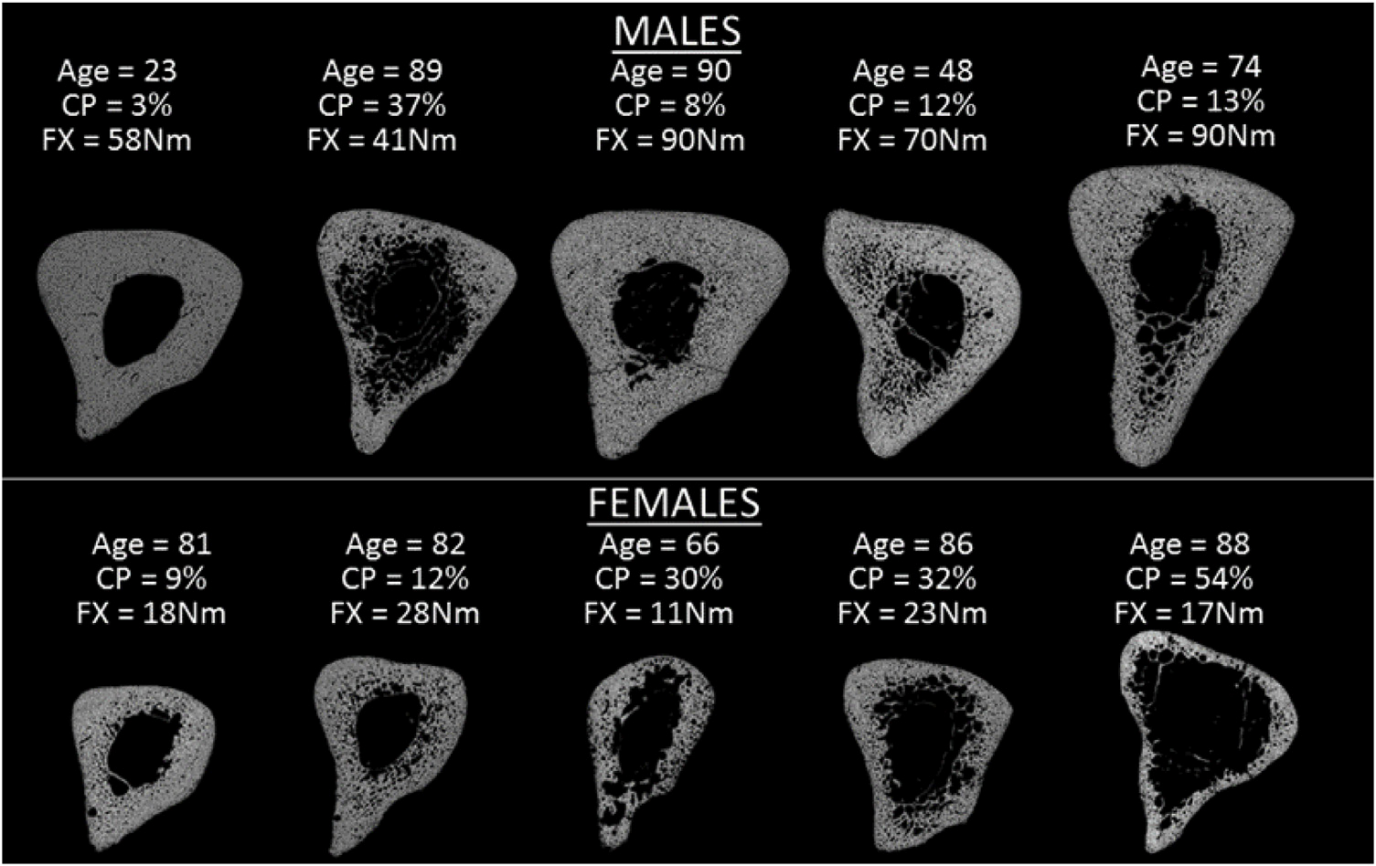
Cross-sectional microtomographic images at the mid-shaft of selected ulna bones [1].

To understand why it is impossible for a patient or operator to know where to place the force probe during MRTA data collection, consider first that the data analysis described in Eqs. (1)–(3) above implicitly assumes that the line of oscillatory force shown in the mechanical model illustrated in Fig. 1 passes through the centroid of the bone, i.e., the point around which the mass of the bone is uniformly distributed. Fig. 7 shows the wide variations in geometric form and patterns of internal osteoporotic destruction of cortical bone tissue at the mid-shaft of the ulna bone. Indeed, Fig. 7 understates this variation as ulnas also vary longitudinally in their axial curvature and diameter. Of course, all of this geometric variation is also obscured by overlying soft tissue.

**Fig. 8:**
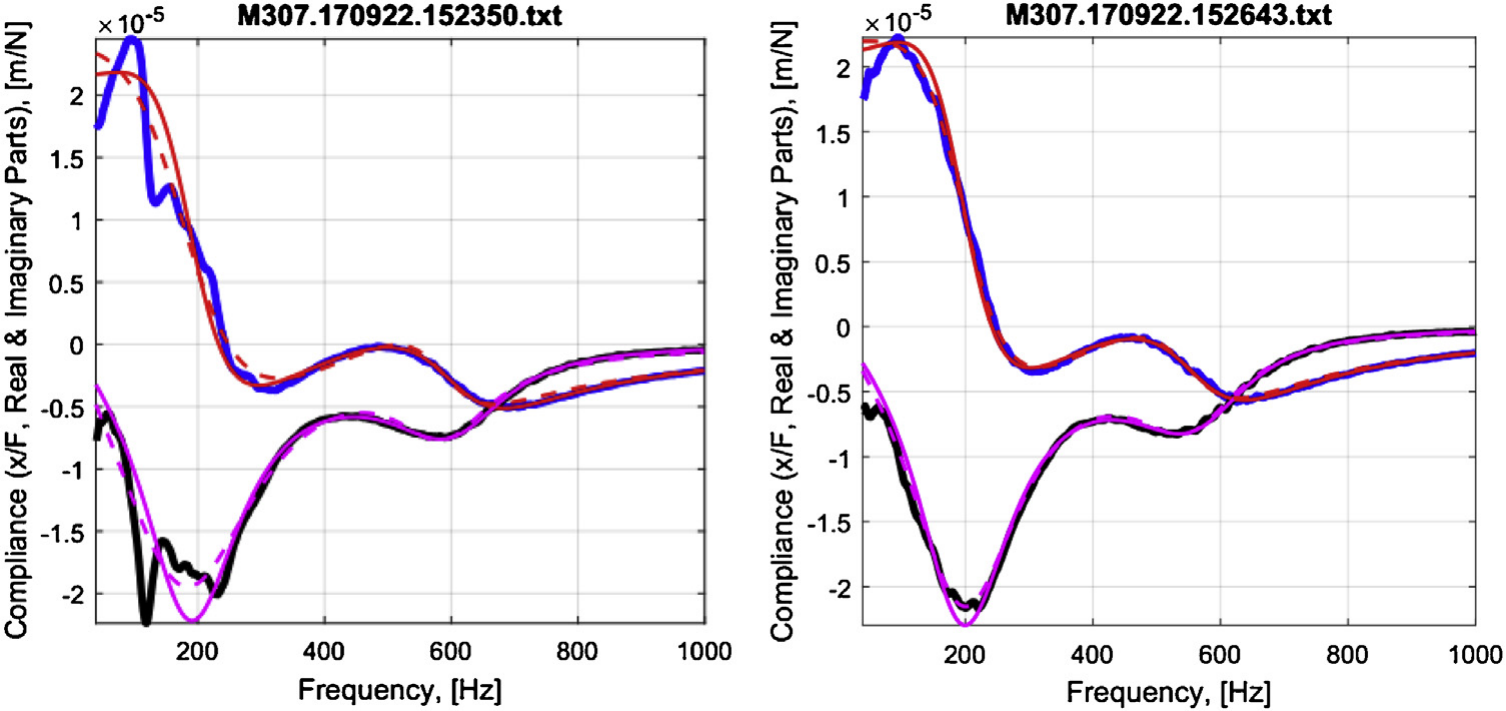
Complex compliance FRFs with (left) and without (right) an extraneous mode of vibration at 121 Hz. Blue = real part of FRF; black = imaginary part of FRF; red = real part of TF; pink = imaginary part of TF; dashed = TF fitted to complex compliance FRF; solid = inverted TF fitted to complex stiffness FRF. Note: the X-axis begins at 40 Hz.

Misplacing the force probe induces modes of vibration other than the pure antero-posterior bending (e.g., medio-lateral bending and torsion) assumed in the derivation of Eq. (1). Introducing additional modes of vibration not included in the seven-parameter model of Eq. (1) has the effect of biasing estimates of the seven parameters, just as an outlying data point biases estimates of the Y-intercept and slope in a linear regression analysis. Fig. 8 illustrates this source of error with two forearm FRFs collected and fitted to TFs 3 min apart. A 4 mm lateral misplacement of the force probe induced an extraneous mode of vibration at 121 Hz that biased the estimate of ulna K_B_ and EI low by 15%.

### Sources of error in MRTA data analysis

The first source of error we encountered in MRTA data analysis related to fits of compliance and stiffness TFs to corresponding compliance and stiffness FRFs. Theoretically, the information in a rational function (y = numerator/denominator) and its inverse (1/y = denominator/numerator) should be the same. However, contrary to theoretical expectation, we observed that estimates of parameter values derived from fits of the complex compliance TF and its inverse (the corresponding complex stiffness TF) to a complex compliance FRF and its inverse (the corresponding complex stiffness FRF) often differed widely from each other. The values of EI calculated from fitting the complex compliance FRF with an extraneous peak in Fig. 7 and its inverse were 32.7 and 27.5 Nm^2^. In our experience, such differences from averages of the paired estimates have ranged up to almost ±200%.

**Fig. 9:**
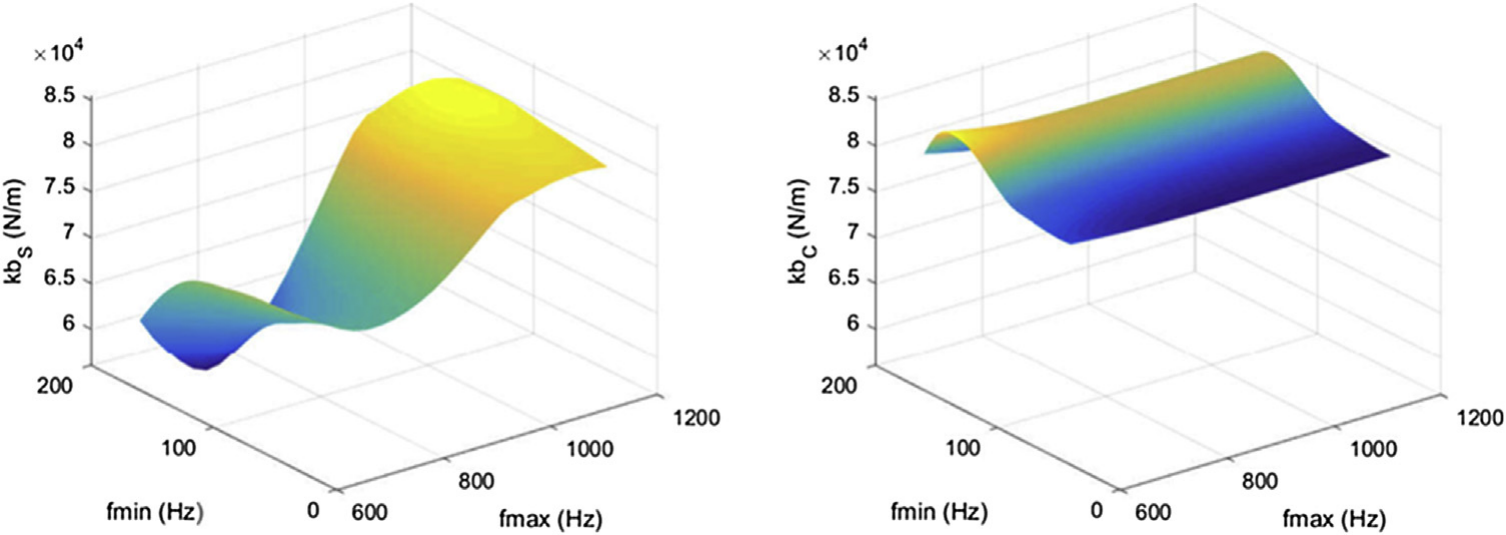
Variation of estimates of ulna bending stiffness from fits of the complex stiffness TF to the complex stiffness FRF (kb_S_, left), and from fits of the complex compliance TF to the complex compliance FRF (kb_C_, right), with the frequency range over which fits were performed. Note: f_min_ = minimum frequency of the fitting frequency range; f_max_ = maximum frequency in the fitting frequency range.

The second source of error we encountered was that estimates of parameter values varied greatly with the frequency range over which the TF was fitted. Fig. 9 illustrates how estimates of ulna bending stiffness varied with the frequency range over which one FRF was fitted.

The third source of error we encountered lay in the selection by previous practitioners of the coefficient of determination (R^2^) to judge the goodness of fit of a TF to a FRF. MRTA practitioners commonly rejected fits to FRFs with R^2^ < 0.90 [16,17]. We found that this threshold and, indeed, this statistic do not discriminate well between widely divergent estimates of parameter values. For example, R^2^ > 0.90 accepted all the FRFs shown in Fig. 6. The values of R^2^ for the fits of TFs to the complex compliance FRFs shown in Fig. 8 were 0.9651 (left) and 0.9960 (right). For the fits of the inverse TFs to the corresponding inverse complex stiffness FRFs, the values of R^2^ were 0.9988 (left) and 0.9996 (right). In our experience testing forearms, we have rarely seen R^2^ as low as 0.95.

In summary, we found that MRTA estimates of model parameter values vary substantially [1] between FRFs collected at closely placed locations, [2] between fits of TFs to stiffness and compliance expressions of a single FRF, and [3] between sub-ranges of frequency in either expression of a single FRF. Complicating this problem, we found that R^2^ does not discriminate well between widely divergent estimates of parameter values.

## Corrections for major sources of error in MRTA

### Corrections for sources of error in MRTA data collection

We avoided one major source of error in MRTA data collection by deferring all effort to measure the mechanical properties of the tibia until after we had mastered measurement of the mechanical properties of the biomechanically much simpler ulna. We avoided another major source of error by removing from the subject all responsibility for positioning the forearm.

We avoided a third major source of error by not asking the operator to try to identify the correct position to place the force probe. Instead, the force probe is initially placed in a known incorrect position maximally eccentric at the mid-shaft. Excitation of the forearm at this position induces an FRF with a large extraneous peak like that shown in Fig. 8. Then FRF data are collected and analyzed at 1 mm increments across the diameter of the ulna, almost all of which positions are also eccentric to some degree. Among them, however, is the position at which the force passes closest to the centroid of the ulna. This procedure exchanged an intractable *a priori* data collection problem for a soluble *post hoc* data analysis problem: recognizing data collected in antero-posterior bending at the centered position. This exchange of problems required the development of an algorithm for recognizing FRF data from the correct site. Before such an algorithm could be developed, however, it required the adoption of certain decision rules for the preference of one estimate of parameter values over many others.

### Corrections for sources of error in MRTA data analysis

#### Decision rules

In order for decision rules for preferring one estimate of model parameter values over others to be widely accepted, they needed to have certain self-evident properties. First, they needed to be generally applicable to all arms. Second, they needed to be non-arbitrary. Third, they needed to yield estimates of parameter values that are physically realizable. Because all of the parameters of the mechanical model illustrated in Fig. 1 are positive, for example, decision rules needed to exclude fits to FRFs that yield negative estimates of parameter values. They also needed to exclude FRFs yielding impossibly high estimates of parameter values. The fourth property of widely acceptable decision rules was that they needed to be objective. Fifth, they needed to be quantitative so that they could be applied by a computer algorithm. Finally, the decision rules needed to yield estimates of ulna bending stiffness similar to estimates obtained by QMT, since QMT is the Gold Standard (i.e., widely accepted) method for measuring bone stiffness. Because the first step in QMT is to remove a bone from the body, this last property required the testing of cadaveric human arms to obtain data with which to discover these decision rules.

#### A more discriminating goodness-of-Fit statistic

It took a long time for the right question to come to mind about the discrepancies between estimates of parameter values from fits of the complex compliance and stiffness TFs to the complex compliance and stiffness FRFs. The right question was, “How big would these discrepancies be in an FRF with perfect 7-parameter data instead of empirically measured data.” To find out, we substituted a plausible set of parameter values into Eq. (3) to calculate the coefficients that would normally be obtained by fitting Eq. (2) to an empirical FRF. We then substituted those coefficients and values of frequency at 1 Hz intervals from 40 to 1200 Hz into Eq. (2) to obtain a synthetic complex stiffness FRF of the same size as an empirical FRF. We then inverted this perfect complex stiffness FRF to obtain the corresponding perfect complex compliance FRF. Then we fitted compliance and stiffness TFs to these perfect FRFs just as we would fit empirical compliance and stiffness FRFs. We found the estimated parameter values from the two fits to be identical and equal to the initial values substituted into Eq. (3). After confirming this with other sets of initial values, we concluded that the discrepancies are a measure of the extent to which an FRF deviates from the 7 parameter model shown in Fig. 1.

**Fig. 10:**
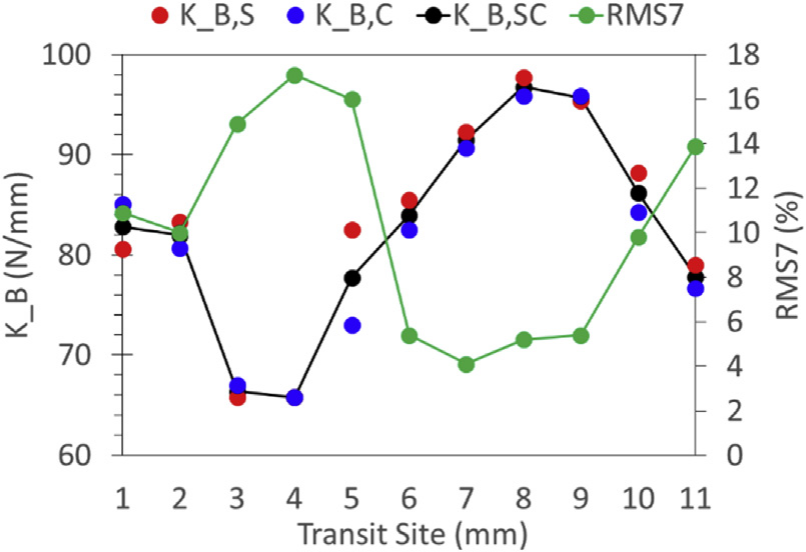
Variation in RMS7 and K_B_ in a transit across a forearm.

Accordingly, we defined the root mean square of the pairwise deviations between the seven parameters normalized to their pairwise means (RMS7) as a candidate goodness-of-fit statistic. We found that RMS7 varied widely (from 0.2% to 90.5%) for FRFs that varied widely in measures of K_B_ but hardly at all in values of R^2^ crowded near 1. For example, values of RMS7 for the FRFs shown on the left and right of Fig. 8, were 13.9% and 5.1%, respectively. Fig. 10 shows how values of RMS7 and K_B_ estimated from the complex stiffness FRF (K_B,S in Fig. 10) and from the complex compliance FRF (K_B,C) varied in one transit across a forearm. Note that the smallest discrepancy between estimates of K_B_ alone occurred at site 4, whereas RMS7 was minimized at site 7.

**Fig. 11:**
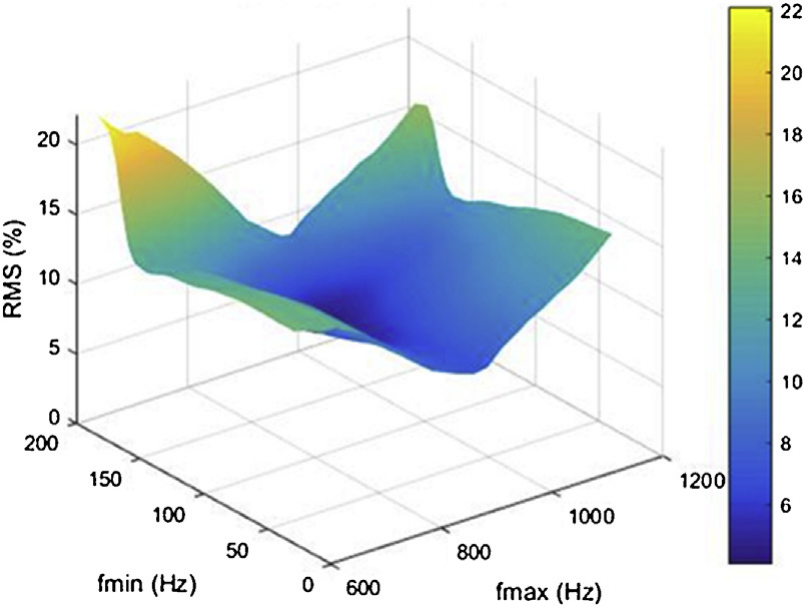
Variation in RMS7 across subranges of frequency in a single FRF.

Fig. 11 shows how values of RMS7 varied across subranges of frequency in complex compliance and stiffness FRFs collected at the same site shown in Fig. 9. Note that RMS7 was minimized when complex compliance and stiffness TFs were fitted to the complex compliance and stiffness FRFs over a particular subrange of frequency.

Two clarifications should be made at this point. First, the values of K_B_ at particular sites such as those plotted in Fig. 10 are those determined in the particular subrange of frequency with the lowest value of RMS7 as shown in Fig. 11. Second, bounds on sub-ranges of frequency are data-driven. The lower bound on the lower end of sub-ranges is the minimum shaker excitation frequency. The type of electromagnetic shaker utilized in the CBMT system applies a constant acceleration to the armature to which the force probe is attached. As a result, the armature’s amplitude of motion is inversely proportional to the square of frequency. Below 40 Hz the amplitude exceeds the physical range of motion provided for the armature within the shaker. The upper bound on the lower end of sub-ranges is the resonant frequency of the bone peak, which, of course, varies from FRF to FRF. Meanwhile, the upper bound on the upper end of sub-ranges is the maximum shaker excitation frequency, which is well above that of the skin peak. For fitting skin peaks with resonant frequencies below about 800 Hz, additional asymptotic data above 1000 Hz are not useful. The lower bound on the upper end of sub-ranges is the resonant frequency of the skin peak. Thus, all sub-ranges of frequency for fitting TFs to FRFs include the range between the bone and skin peaks, and thereby capture the influences of model parameters on the locations and shapes of those resonances. Whether low values of RMS7 indicate *correct* values of K_B_ remained to be determined, however.

## Validation of CBMT by QMT

As reported [1] in the companion to this article, the correctness of the above innovations was validated by comparing noninvasive CBMT measurements of K_B_ in 35 cadaveric human arms to measurements by QMT in the ulnas excised from those arms. Male and female arm donors ranged widely in age (17 to 99 years) and body mass index (13–40 kg/m^2^). Because QMT force-displacement curves were typically nonlinear, QMT measurements of bending stiffness were recorded as the peak tangent stiffness. Discrepancies between CBMT and QMT measurements over all 35 arms were minimized by requiring RMS7 ≤ 9% in fits of TFs to FRFs. After correcting for a fixed bias thought to be due to ligamentous binding of the ulna to the radius in the intact arm, the linear relationship between CBMT and QMT measurements of ulna EI approximated the identity line (CBMT = 1.00 QMT, R^2^ = 0.99). Linear relationships (y = b_0_ + b_1_x) between ulna bending strength measured by QMT and ulna EI measured by CBMT (adjusted) and QMT (both R^2^ = 0.99) were indistinguishable from one another (b_0_ = 0, both p ≥ 0.24; b_1_ = 1.29 ± 0.02 (QMT) vs 1.28 ± 0.03 (CBMT), p ≥ 0.80).
